# Four-word lexical bundles in Chinese-English consecutive interpreting—A comparative study between professionals and trainees

**DOI:** 10.3389/fpsyg.2022.1005532

**Published:** 2022-10-13

**Authors:** Fang Tang, Shuzhen Jiang

**Affiliations:** ^1^Centre for Translation Studies, Guangdong University of Foreign Studies, Guangzhou, China; ^2^School of Interpreting and Translation Studies, Guangdong University of Foreign Studies, Guangzhou, China

**Keywords:** four-word lexical bundles, characteristics, reasons, professionals, trainees, consecutive interpreting

## Abstract

As comparative studies on lexical bundles between professional interpreters and trainees are pedagogically significant but rare, this experimental study initiates a comparison on the product and process of four-word lexical bundles in Chinese-English consecutive interpreting between these two groups. Frameworks regarding the structure and strategy of lexical bundles are established to analyze the product of lexical bundles produced by these two groups, and data including interpreters’ interpreting products, notes as well as retrospection and interviews are collected to analyze their process of producing lexical bundles. The results show that the types (Type) and frequencies (Token), except diversity (TTR), of lexical bundles with the “noun and/or prepositional phrase fragments” structure and/or the “equivalence” strategy from professionals are significantly higher than those from trainees. Reasons for inter-group similarities and differences in structural and strategical distributions (product) and strategy adoption (process) are also analyzed. Based on the established interpreting-tailored lexical bundle frameworks, this comparative study presents and explains similarities and differences between professionals and trainees and implies suggestions for the training and learning of interpreting.

## Introduction

Lexical bundles have long been researched in the speaking and writing of natives or second language learners (Altenberg, 1998; Biber et al., 1999, 2004; Li, 2004; Wei, 2004; Biber and Barbieri, 2007; Wang and Wang, 2008; Biber, 2009; Arnon and Snider, 2010). However, lexical bundles produced by interpreters, possibly a group with the highest second language proficiency, are almost out of the scope of academics in second language acquisition. Such marginalization may be explained from two angles. Firstly, interpreting, though an ancient activity, is in lack of theoretical assumptions, since it has been studied as an independent research object for just decades (Salevsky, 1993; Gile, 1994). Secondly, the implementation of large-scale research on interpreting is highly difficult mainly due to the unavailability of abundant qualified interpreters.

Nonetheless, interpreting studies, especially corpus-based interpreting studies, have witnessed profound development in recent decades (Pöchhacker and Shlesinger, 2002; Pöchhacker, 2016; Wang and Fu, 2020). Firstly, interpreting, as a major, has fostered quite a few qualified subjects and researchers, as an increasing number of Master of Translation and Interpreting (MTI) programs are launched in universities and the enrollment of trainees is growing (Mu, 2020). Secondly, interpreting, as a profession, is gaining importance in more diverse social settings, from international conferences to diplomatic interactions, from media broadcasting to business negotiation, and from legal consultation to medical diagnosis or treatment. Thirdly, interpreting, as a research field, has been equipped with continuously evolving technologies, i.e., recording, transcribing, tagging, and searching technologies for analyzing interpreting corpora. To sum up, the increasing number of interpreting trainees, the varying application of interpreting practices and the unceasing upgrade of research approaches all pave the way for the deeper and broader exploration of lexical bundles in interpreting.

Lexical bundles, in this study, are defined as recurrent multi-word sequences without considering their idiomaticity and structural status (Biber et al., 1999, p. 990). Frequency, as the only screening criterion, tends to identify lexical bundles unnoticed by perceptual salience (Biber et al., 2004, p. 376). Those lexical bundles are worthy of description and explanation, for they may deepen researchers’ understanding of the product of lexical bundles and the process of producing lexical bundles in interpreting. This study, based on a self-built interpreting corpus, aims to improve the investigation frameworks of lexical bundles and provide suggestions for interpreting pedagogy *via* comparing the product and process of lexical bundles adopted by professionals and trainees.

## Literature review

The length, taxonomy, and producer of lexical bundles are recurring topics of interest in interpreting. The length of lexical bundles ranges from one word to six words; the taxonomies of lexical bundles are illustrated from the perspectives like structure, function, and strategy; and the producers of lexical bundles involve professionals and trainees at different stages.

“Lexical bundles of any length can be analyzed” (Biber, 2009, p. 282). The one-word and two-word lexical bundles (e.g., well, you know, I mean) are mainly manifested as pragmatic markers, which contain almost no propositional information and can be deleted without influencing the original meaning of an utterance (Brinton, 1996; Li, 2004; Wang and Wang, 2008). The three-word to six-word lexical bundles, as multiple clause constituents, can be put in any position of a clause according to their functions, contributing to the exploration of linear organization of utterances: thematic springboard (frame + onset + stem) and prepositional core (rheme + tail + transition) (Altenberg, 1998, p. 109; Shao, 2018a,b, p. 51, p. 72). Four-word lexical bundles are reckoned to be more significant than lexical bundles of other lengths, for their structures and meanings are relatively more complete than 1/2/3-word lexical bundles (Biber and Barbieri, 2007; Chen and Baker, 2010) and their frequencies are generally higher than 5/6-word lexical bundles (Shao, 2018a, 2018b). Besides, among previous investigations on multi-word lexical bundles in the written and spoken registers, most studies focus on four-word sequences (e.g., Biber and Barbieri, 2007; Chen and Baker, 2010). In order to fully use previous findings, this study narrows the investigation scope and focuses also on four-word lexical bundles in interpreting.

The taxonomies of lexical bundles have been established based on their structure, function, and strategy. The structure of lexical bundles is divided into “verb phrase fragments,” “independent clause fragments,” and “noun phrase and prepositional phrase fragments” (Biber et al., 2004). Their functions are classified into “stance expressions,” “discourse organizers,” “referential expressions,” and “special conversational functions” (*ibid.*). Their strategies are categorized into “equivalence,” “addition,” and “shift” by Xu and Li (2021). Although the three taxonomies, especially the former two, have already been applied to quite a few interpreting studies (e.g., Cortes, 2004; Partington and Morley, 2004; Li, 2016, 2017; Shao, 2018a,b; Xu and Li, 2021; Li and Halverson, 2022), their validity is still questionable. For one thing, their applicability in the field of interpreting research is doubtable. The taxonomies regarding the structure and function are founded based on data from the spoken (conversation and classroom teaching) and written (textbooks and academic prose) registers. Considering the specificity of interpreting, it might not be sensible to apply them directly into the investigation of lexical bundles in the interpreting register. For another, the scope and level of comparison are vague (e.g., Xu and Li, 2021). The strategical taxonomy of lexical bundles, no longer confined to the spoken and written registers, has been extended to the interpreting register (e.g., Xu and Li, 2021; Li and Halverson, 2022). Its establishment is based on translational relationships between ST and TT of professional interpreters, yet within those comparisons, the scope (“the original sentence vs. the corresponding interpreted sentence” or “the original message vs. the target sentence”) and the level (lexical, semantic, or pragmatic level) of comparison have never been clearly stated. In view of these two aspects, this study first scopes the extent of application and then specifies the range and level of comparison between ST and TT, so as to customize a series of classification frameworks for lexical bundles in interpreting.

Research has given heed to the lexical bundles produced by professional interpreters or trainees, but a comparison between them fails to garner attention. For professionals, Plevoets and Defrancq (2018, p. 23) found that the more formulaic sequences they used, the less cognitive stress they experienced. Specifically, cognitive pressure tends to be alleviated by the prediction, storage, and production of lexical bundles (Li and Wang, 2012). In addition, the linguistic characteristics (structure, function and strategy) and strategy adoption of professionals’ lexical bundles have attracted attention (Henriksen, 2007; Shao, 2018a, b; Li and Zhang, 2020; Xu and Li, 2021; Li and Halverson, 2022). As for structure, Henriksen (2007, p. 9, 18) argued that the homogeneity of the interpreting products is enhanced as multiple ideas are “transposed into one target text formula” and “interpreters tend to borrow formulaic phrases from colleagues.” For function, Li and Halverson (2022) explored the distribution of four discourse functions (stance expressions, discourse organizers, referential expressions, and special conversational functions) and their corresponding patterns in interpreting (partial and complete equivalence, shift, and addition). In terms of strategy, Li and Zhang (2020) found that the complete or partial addition of lexical bundles achieves three goals: to lengthen the target text, to reuse the lexical bundles, and to complete the political terms. Those studies are good examples of describing the linguistic characteristics and strategy adoption of lexical bundles from different perspectives, while the reasons behind them are much less discussed. The present study aims to not only explore the characteristics and strategies of lexical bundles but also figure out the reasons behind them.

For trainees, cognitive pressure, interpreting competence, and pedagogical implications related to lexical bundles have piqued the interest of academics. Firstly, their cognitive pressure can be alleviated by certain structures, functions, and strategies of lexical bundles. For structure, the “noun phrase and prepositional phrase fragment” lexical bundles are proved to be the greatest alleviation of cognitive strain in interpreting (Li, 2017, p. 93). For function, relatively fixed and frequently used lexical bundles of stance, discourse organizing and reference functions contribute to the automation of outputting and win more time for interpreters to focus on the following information (Li, 2016). For strategy, pragmatic markers, which have the effects of choosing stances, explicating and enriching pragmatic information, are utilized as an unmarked strategy without consuming too much cognitive effort in interpreting (Li and Zhao, 2019, p. 37). Secondly, interpreting competence, reflected by different levels of interpreting scores (four levels: excellent, good, fair, and poor scores), is closely related to the linguistic features of lexical bundles. Specifically, a higher level of interpreting scores corresponds to more tokens in any of the three structures of lexical bundles (verb phrase fragments, dependent clause fragments or noun phrase and prepositional phrase fragments) (Li, 2017, p. 93). Besides, the relationship between the levels of interpreting scores and the type-token-ratio (TTR) of lexical bundles is U-shaped, signifying that the two are negatively correlated before a critical point (the level of fair scores) and positively correlated after it (*ibid*). Moreover, the relationship between interpreting competence (three levels: high, medium, and low marks) and the frequency of correct or incorrect lexical bundles is explored, and the incorrect ones are further classified and analyzed through a large-scale experiment and interviews with a small group of trainees (Wang and Huang, 2011, 2013). Thirdly, pedagogical implications of lexical bundles in interpreting have been affirmed theoretically and empirically. Theoretically, the opinion that an unawareness of lexical bundle training diminishes interpreting quality, including fluency, idiomaticity, and accuracy, was held by Song et al. (2014), but their examples are limited and the conclusions are subjective. Empirically, a one-semester experiment with multiple comparison tests for two classes of trainees confirmed that the training of lexical bundles, at least 2 months, significantly improved trainees’ accuracy rate in interpreting (Wang, 2012, p. 50). Similar to studies focusing on professionals, only the linguistic characteristics of lexical bundles from trainees are well described; thus, this study will further explain reasons behind those characteristics. Unlike researches on professionals, strategy adoption of trainees is rarely studied, possibly for trainees’ interpreting proficiency is relatively lower and might have less reference value for other interpreters. However, a systematic comparison requires description and exploration on strategy adoption of both professionals and trainees; thus, the present study not only analyzes trainees’ strategy adoption but also explores reasons behind it. By doing so, a comparative study on linguistic features and strategy adoption between professionals and trainees can be achieved, providing directions for trainees in learning and trainers in teaching.

As shown above, although investigations into the linguistic features or strategical adoption of lexical bundles produced by professional interpreters or trainees have been conducted, basically no comparative study between them can be found. This may be attributed to the difficulty of subjects inviting and the complexity of research procedures. The present study, overcoming the limitations of previous studies, intends to compare the linguistic distributions (product) and strategy adoption (process) of lexical bundles between these two groups by exploring the following four questions. The former two questions are product-oriented, while the latter two are process-oriented.

1. What are the characteristics of the structural and strategical distributions of four-word lexical bundles produced by professional interpreters and trainees?
2. What are the similarities and differences in the structural and strategical distributions of four-word lexical bundles produced by professional interpreters and trainees?
3. What are the reasons for the strategies of four-word lexical bundles produced by professional interpreters and trainees?
4. What are the similarities and differences in the reasons for the strategies of four-word lexical bundles produced by professional interpreters and trainees?

## Materials and methods

### Participants

Nine professional interpreters and nine interpreting trainees, all with Chinese as their A language and English as B language, were invited to participate in this experiment voluntarily and anonymously. The professional group included those with an average of 4 years of working experience, among which five were interpreter trainers and four were in-house interpreters. The trainee group was all postgraduates from a university in Hong Kong. Without any working experience, those trainees had received one-semester systematic interpreting training.

### Procedure

Data were collected and processed *via* the following steps: (1) Preparation: After being briefed with the procedure of the experiment, the participants had 10 min to know about the background information and terminology of the Chinese speech. (2) Warm-up exercise: The participants were asked to listen to a relevant recording to get familiar with the pronunciation and tone of the speaker and could adjust the volume according to their own preference before the formal interpreting task began. (3) Consecutive interpreting (CI): All participants did the CI task segment by segment with their interpreting products being recorded. They were allowed to take notes during the interpreting process. (4) Retrospection and interviews: When interpreting finished, the participants reflected on the interpreting process based on the transcribed source speech and interpreting recordings. Meanwhile, researchers may ask questions to further explore the interpreting process. (5) Data collection: Recordings of the interpreting products, retrospection, and interviews of all participants as well as their interpreting notes were collected for later analysis. (6) Transcription and annotation: All the recordings were transcribed with those disfluencies in the interpreting products being annotated (e.g., <p> for silent pauses, <uh> for hesitations, and <~> for stretched pronunciations).

### Corpus

The corpus includes one Chinese text as ST, 18 English texts as TT. The ST is a transcribed impromptu speech, comprising a total of 1,566 characters and lasting 6 min and 50 s. The speech was delivered by one of China’s former Minister of Education as an answer to a question about education reform raised by a journalist at a press conference. The TT, 18 transcribed recordings from the two groups performing consecutive interpreting assignments, is stored in two sub-corpuses, namely the Chinese-English Consecutive Interpreting Corpus for Professionals (CCICP) and the Chinese-English Consecutive Interpreting Corpus for Trainees (CCICT). Both CCICP and CCICT consist of one Chinese text and nine English texts while CCICP totals 9,731 English words and CCICT totals 9,697 English words.

### Retrieving and screening of lexical bundles

AntConc 3.5.9 is adopted to extract lexical bundles from the two sub-corpora (CCICP and CCICT) respectively. Firstly, the maximum and minimum of *n*-gram size are set at 4, meaning that retrieved lexical bundles are four-word units. Secondly, the minimums of frequency and range are set at 4 and 1 respectively, indicating that extracted lexical bundles appear four or more times in at least one text. The frequency cut-off (four times per 10,000 words equals 400 times per million words) in this study is much higher than that in Biber et al. (2004, p. 376), who took a frequency of 40 times per million words. The more conservative frequency setting-off in this study can help reduce occasionality in a relatively smaller corpus. The minimum range was set at 5 by Biber et al. (2004, p. 376), which was somewhat arbitrary, to “guard against idiosyncratic uses by individual speakers or authors” (*ibid*). In this study, the ST is a speech about education reform (a non-specialized topic) and the TT contains no idiosyncratic uses.^1^ Therefore, the range cut-off setting at 1 in the present study can ensure a more comprehensive investigation, which may also reveal the interpreters’ personal preference in using lexical bundles.

Retrieved lexical bundles require manual screening. Lexical bundles containing the name of a person or cross-sentence structures (e.g., rural areas. And we…) are screened out. Another two types, those with incomplete structures (e.g., behalf of the government, and at the same) and those with disfluencies (including pauses, hesitations, repairs, etc.), which were filtered out in previous studies (De Cock, 1998; Wei, 2004; Wang and Huang, 2013; Li and Zhao, 2019; Xu and Li, 2021), are retained in this study. On the one hand, incomplete structures are not necessarily insignificant (Biber et al., 1999, 2004, p. 990, p. 371). For instance, **(on/on the/at)** **behalf of the government** reflects interpreters’ lack of mastery of grammar; **and at the same** **(time/place/age)** shows the flexibility of collocation in one lexical bundle. Thus, retaining lexical bundles with incomplete structures in this study may be instructive for interpreting training. On the other hand, disfluencies, though reducing the fluency of lexical bundle output, still reflect crucial information unmarked by previous studies which deleted them directly. Eliminating lexical bundles with disfluencies widens the frequency gap between professionals and trainees, for the latter tends to produce more lexical bundles with disfluencies. Instead, scientific tagging ways of paralinguistic information (such as hesitations, pauses, etc.) and repairs (including error repairs, explicitation repairs, precision repairs, synonymy repairs, restart repairs, and repetitions) have been discussed (Zou and Wang, 2014; Zhang, 2015; Tang, 2020a, 2020b) and allowed critical information to surface through disfluencies in lexical bundles. For example, hesitations symbolize mind-blanking or buffer strategies (Tang, 2020c, p. 42). Following the systematic tagging approaches, this study annotates those disfluencies in interpreting products, a methodological advance in identifying features of lexical bundles from a more comprehensive perspective.

### Types and definitions of structures and strategies of lexical bundles

This study analyzes lexical bundles from two aspects: structure and strategy. As for structure, Biber et al. (2004) classified lexical bundles from classroom teaching and textbooks into three main types: “verb phrase fragments,” “dependent clause fragments,” and “noun phrase and prepositional phrase fragments.” Most of the structural types and related definitions, suitable for lexical bundles in this study, are retained while slight modifications are made to the third type. Except providing it with a more concise name “noun and/or prepositional phrase fragments,” the third type is redefined as “lexical bundles incorporating only noun and/or prepositional phrase fragments” to avoid possible mistakes in classification (see Table 1).

**Table 1:** Definitions and examples of structural and strategical types of lexical bundles.

Structural Types	Definitions	Examples
Verb phrase fragments	Lexical bundles incorporate verb phrase fragments.	was the Teachers’ Day and now we are
Dependent clause fragments	Lexical bundles incorporate dependent clause fragments.	that is why I as I said just
Noun and/or prepositional phrase fragments	Lexical bundles incorporate only noun and/or prepositional phrase fragments.	a very important speech the development of the
Strategical Types	Definitions	Examples*
Equivalence	Lexical bundles in TT are equivalent to messages in ST.	ST: 我们就是要 TT: **what we need to do is to**
Addition	Lexical bundles in TT are additional to messages in ST.	ST: 一个最重要的措施呢 TT: one of the most important way **to improve the quality of the rural teachers’** team
Shift	Lexical bundles in TT are substitutive to messages in ST.	ST: 办好教育 TT: **the development of the** Chinese education
Equivalence + Shift	Lexical bundles in TT incorporate both “equivalence” and “shift” strategies when being compared to messages in ST.	ST: 有一系列的措施 TT: **have a lot of** measures
Equivalence + Addition	Lexical bundles in TT incorporate both “equivalence” and “addition” strategies when being compared to messages in ST.	ST: 不低于当地 剬务员的 TT: **that of the civil servants**
Shift + Addition	Lexical bundles in TT incorporate both “shift” and “addition” strategies when being compared to messages in ST.	ST: 绩效考核 TT: **This is a very** important measure
Equivalence + Shift + Addition	Lexical bundles in TT incorporate “equivalence”, “shift,” and “addition” strategies when being compared to messages in ST.	ST: 但是面对着新的形势 TT: **And now we are** in a new era

*Explanations of these lexical bundles will be elaborated in the section “Comparing the process of lexical bundles between professionals and trainees.”

In terms of strategy, Xu and Li (2021) put lexical bundles in simultaneous interpreting products into three categories: “equivalence,” “addition,” and “shift.” These three one-strategy patterns, applicable for lexical bundles in this study, are retained. Moreover, four multi-strategy patterns, namely “equivalence + shift”, “equivalence + addition”, “shift + addition” and “equivalence + shift + addition” are newly added in this study.

The definitions of strategies for lexical bundles proposed by Xu and Li (2021) still have room for improvement. First, their definitions do not clarify the scope of comparison. If a lexical bundle in the last sentence or paragraph in TT corresponds to the information in the first sentence or paragraph in ST, is it still an “equivalence” strategy? Second, their definitions do not clarify the level of comparison. If the words in a lexical bundle in TT correspond to the characters of a specific message in ST, but the semantic meaning of the lexical bundle as a whole does not correspond to the message,^2^ is it still an “equivalence” strategy? Both questions will be solved with extended definitions in this article. To be more rigorous, the comparison scope is set as “the original sentence vs. the corresponding interpreted sentence” and the comparison level “semantic meaning^3^,” including the semantic meaning of the single substantive word in one lexical bundle as well as the lexical bundle itself as a whole. With comparison scope and level being determined, strategical types are clearly defined and exemplified in Table 1.

## Results

This study explores the distributions of four-word lexical bundles of interpreters from the perspective of structure and strategy. Structural distributions show certain features of the two groups’ interpreting products, and strategical distributions reveal the relationships between ST and TT mediated by the two groups. The inter-group similarities and differences in their structural and strategical distributions are analyzed below.

### Structural distributions of lexical bundles from professionals and trainees

The distribution of structural patterns of four-word lexical bundles from professionals and trainees is reported in Table 2. “Type^4^” stands for the number of types of lexical bundles produced by interpreters, “Token” the frequencies of lexical bundles, and “Type-Token Ratio (TTR)^5^” the diversity of lexical bundles.

**Table 2:** Structural distributions of four-word lexical bundles from the two groups.

	Professionals			Trainees			U (*p*)		
	Type (%)	Token (%)	TTR	Type (%)	Token (%)	TTR	Type	Token	TTR
Verb Phrase Fragments	30 (25.6)	158 (22.5)	0.190	18 (25)	113 (23.7)	0.159	26.000 (0.195)	22.500 (0.111)	36.000 (0.691)
Dependent Clause Fragments	17 (14.5)	94 (13.4)	0.181	11 (15.3)	71 (14.9)	0.155	38.000 (0.821)	35.000 (0.626)	31.000 (0.400)
Noun and/or Prepositional Phrase Fragments	70 (59.8)	451 (64.2)	0.155	43 (59.7)	292 (61.3)	0.147	7.000^**^ (0.003)	8.500^**^ (0.005)	36.000 (0.691)

U: Mann–Whitney rank-sum test.

** *p* < 0.01.

The results show that (1) the order of lexical bundle types (Type) and frequencies (Token) of both groups is: noun and/or prepositional phrase fragments > verb phrase fragments > dependent clause fragments; (2) the rank of lexical bundle diversity (TTR) of the two groups is: verb phrase fragments > dependent clause fragments > noun and/or prepositional phrase fragments; (3) the types (Type), frequencies (Token), and diversity (TTR) of the three fragments’ lexical bundles from professionals are higher than those from trainees. For further observations, this study employs the Mann–Whitney *U* test to check if any statistical significance exists. Significant differences (*p* < 0.01) between the two groups are found in the types (Type) and frequencies (Token) of lexical bundles with “noun and/or prepositional phrase fragments,” indicating that the types and frequencies of lexical bundles with “noun and/or prepositional phrase fragments” from professionals are significantly higher than those from trainees.

### Strategical distributions of lexical bundles from professionals and trainees

The distribution of strategical patterns of four-word lexical bundles from professionals and trainees is illustrated in Table 3. The results show that (1) the strategical pattern with the highest types (Type) and frequencies (Token) is “equivalence,” while strategical patterns with the least types (Type) and frequencies (Token) are “shift + addition” and “equivalence + shift + addition,” either for professionals or trainees; (2) the diversity (TTR) of strategical patterns from professionals, except “addition,” is higher than that from trainees; (3) the types (Type) and frequencies (Token) of “shift + addition” and “equivalence + shift + addition” patterns from professionals are lower than those from trainees, while the diversity (TTR) of the two patterns is the opposite; (4) the types (Type) and frequencies (Token) of “equivalence,” “shift,” “addition,” “equivalence + shift,” and “equivalence + addition” patterns from professionals are higher than those from trainees. Additionally, the Mann–Whitney *U* test is used to further explore whether the two groups’ data differ significantly or not. Significant differences between these two groups are identified in the types (Type, *p* < 0.01) and frequencies (Token, *p* < 0.05) of the “equivalence” pattern, indicating that the types and frequencies of lexical bundles using the “equivalence” strategy from professionals are significantly higher than those from trainees.

**Table 3:** Strategical distributions of four-word lexical bundles from the two groups.

	Professionals			Trainees			U (*p*)		
	Type (%)	Token (%)	TTR	Type (%)	Token (%)	TTR	Type	Token	TTR
Equivalence	97 (82.9)	430 (61.2)	0.226	57 (79.2)	282 (59.2)	0.202	7.000^**^ (0.003)	13.500^*^ (0.017)	38.000 (0.825)
Shift	19 (16.2)	37 (5.3)	0.514	12 (16.7)	35 (7.4)	0.343	35.500 (0.653)	40.000 (0.964)	34.000 (0.553)
Addition	40 (34.2)	75 (10.7)	0.533	19 (26.4)	33 (6.9)	0.576	22.500 (0.108)	19.500 (0.062)	27.500 (0.191)
Equivalence + Shift	30 (25.6)	66 (9.4)	0.455	17 (23.6)	44 (9.2)	0.386	26.000 (0.193)	19.500 (0.058)	34.000 (0.561)
Equivalence + Addition	33 (28.2)	89 (12.7)	0.371	22 (30.6)	61 (12.8)	0.361	24.500 (0.154)	19.500 (0.061)	28.000 (0.262)
Shift + Addition	5 (4.3)	5 (0.7)	1	8 (11.1)	17 (3.6)	0.471	31.500 (0.390)	31.500 (0.390)	38.000 (0.804)
Equivalence + Shift + Addition	1 (0.9)	1 (0.1)	1	2 (2.8)	4 (0.8)	0.5	31.500 (0.270)	31.000 (0.248)	32.000 (0.301)

U: Mann–Whitney rank-sum test.

* *p* < 0.05;

** *p* < 0.01.

## Discussion

The comparison of the product (structural and strategical distributions) and process (strategy adoption) of lexical bundles between professionals and trainees is elaborated in this section with reasons for the similarities and differences in the two groups’ product and process being explained in detail.

### Comparing the product of lexical bundles between professionals and trainees

#### Reasons for similarities and differences in structural distributions of lexical bundles

The types (Type: 14.5% for P^6^; 15.3% for T) and frequencies (Token: 13.4% for P; 14.9% for T) of “dependent clause fragments” lexical bundles are the smallest in both professionals’ and trainees’ output. Dependent clauses are used less in speaking than in writing (O’Donnell, 1974, p. 103). The interpreting register is similar to the spoken register in terms of output immediacy, leading to the fact that the interpreting register is less likely to contain many dependent clauses. Therefore, “dependent clause fragments” lexical bundles are fewer than the other two fragments’ lexical bundles in interpreting output.

“Verb phrase fragments” lexical bundles have the highest diversity (TTR) in both groups’ output (P: 0.190; T: 0.159). The key element of “verb phrase fragments” is verbs. The former’s diversity can be reflected by the latter’s in two aspects. First, verbs are usually advised not to be recorded in notes. Since “relation^7^” is more likely to be deduced by “argument^8^” (Ji, 1996, p. 56; Wang and Zhou, 2014, p. 117), verbs tend to be omitted in notes, with nouns or pronouns being noted down to remind interpreters of which verbs can be used. Alternatively, verbs are recommended to be written down as symbols in notes. One symbol may represent the meaning of multiple synonyms, i.e., the symbol “♡” can mean “want,” “wish,” “desire,” or “hope for” (Gillies, 2017, p. 103). The two recommended methods of remembering verbs in interpreting may make TT deviate a little from ST, but minor deviations do not influence interpreting quality. The two suggestions indicate that the interpreting of verbs is relatively more flexible, that is, less limited by notes or ST, which contributes largely to the high diversity of verbs and then to the high diversity of “verb phrase fragments.” Therefore, the diversity of “verb phrase fragments” lexical bundles is higher than the other two fragments’ lexical bundles.

The types (Type: 59.8% for P; 59.7% for T) and frequencies (Token: 64.2% for P; 61.3% for T) of “noun and/or prepositional phrase fragments” lexical bundles are the largest in these two groups’ output. Significant inter-group differences are found (*p* < 0.01) in the types (Type) and frequencies (Token) of “noun and/or prepositional phrase fragments” lexical bundles. For the similarities, the nouns and pronouns, the key elements of “noun and/or prepositional phrase fragments,” are considered as “argument” to be noted down (Ji, 1996, p. 56; Wang and Zhou, 2014, p. 117), increasing the opportunity of expressing them out in interpreting. Thus, the types and frequencies of “noun and/or prepositional phrase fragments” lexical bundles are augmented in both groups. For the differences, compared with trainees, professionals with more interpreting experience can memorize, either in mind or by notes, and interpret significantly more types and frequencies of “argument.” And professionals are also proved to use much more “addition” strategies than trainees, further boosting the possibility of producing more nouns and/or pronouns and widening the gap of the types and frequencies of “noun and/or prepositional phrase fragments” lexical bundles between professionals and trainees (see the section “Addition”).

#### Reasons for similarities and differences in strategical distributions of lexical bundles

The two groups present different distributions of major strategical patterns. The distribution of the strategical patterns of the professional group (Token: equivalence > addition > shift) confirms the conclusions of Xu and Li (2021) as well as Li and Halverson (2022), while that of the trainee group (Token: equivalence > shift > addition) shows some differences, a new finding in the present study.

Professionals, corroborating the finding of Wang and Li (2015), use the strategy “addition” more frequently than trainees (Token: *p* = 0.062), whereas trainees prefer the strategy “shift” (Token: 7.4%) to “addition” (Token: 6.9%). They tend to find alternatives due to the lack of an exact memory of the original message.

**Figure 1:**
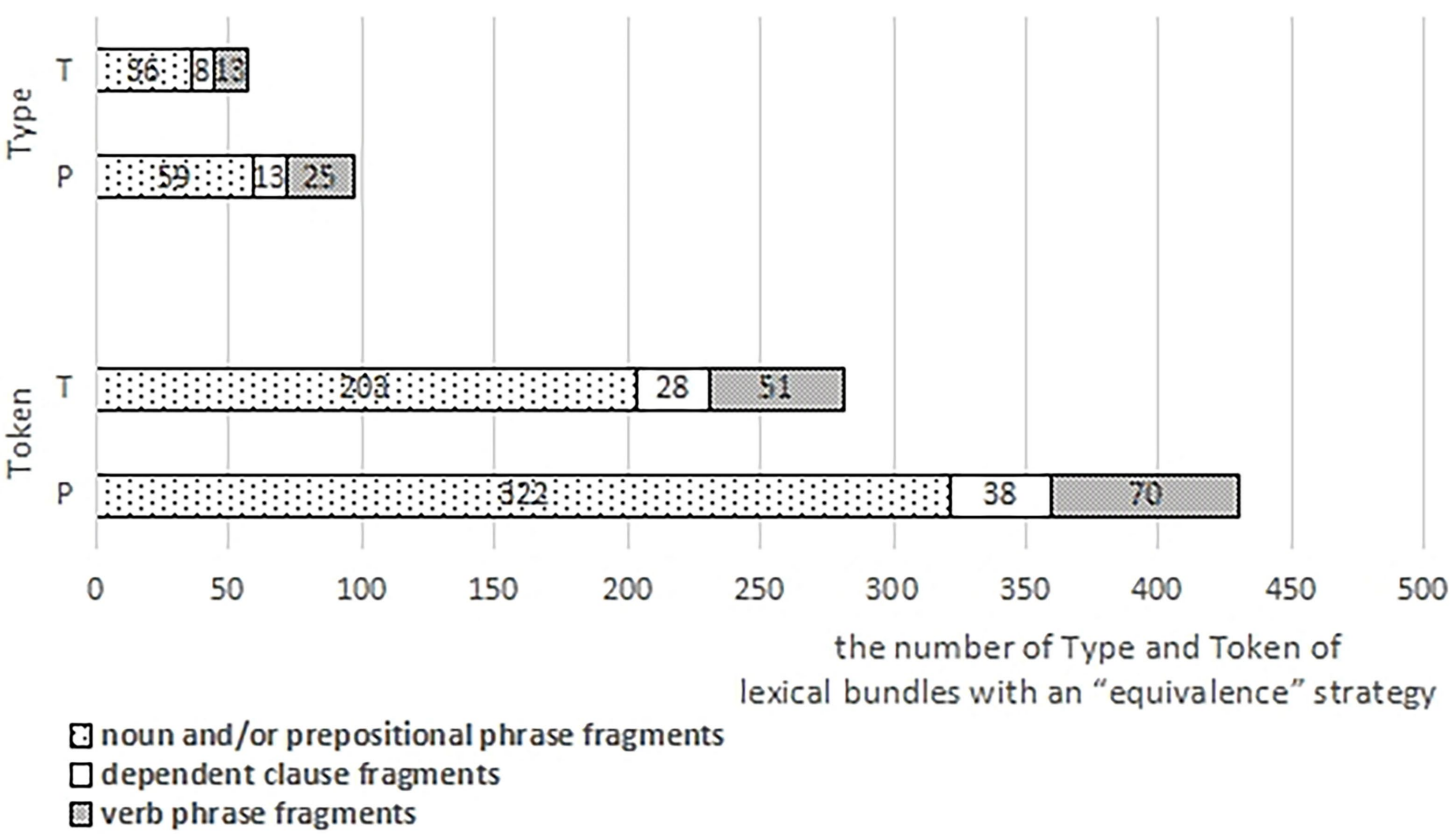
The relationship between the strategy (Equivalence) and structures (Three Fragments) of lexical bundles from the two groups.

The types (Type) and tokens (Token) of lexical bundles with an “equivalence” strategy from professionals are significantly higher than those from trainees. Strategical distributions may have relationships with structural distributions. As is shown in Figure 1, most lexical bundles with an “equivalence” strategy from both groups belong to the structure of “noun and/or prepositional phrase fragments” (Type: 60.82% for P; 63.16% for T; Token: 74.88% for P; 71.99% for T). Hence, the significant inter-group differences in the types and frequencies of lexical bundles with an “equivalence” strategy (see the section “Strategical distributions of lexical bundles from professionals and trainees”) may be largely resulted from those of the “noun and/or prepositional phrase fragments” lexical bundles (see the section “Structural distributions of lexical bundles from professionals and trainees”). Specifically, the more the “noun and/or prepositional phrase fragments” are written down or expressed out, the more “equivalence” strategies tend to be adopted, contributing to the types and frequencies of lexical bundles using this strategy.

Comparing the discussion between the sections “Reasons for similarities and differences in structural distributions of lexical bundles” and “Reasons for similarities and differences in strategical distributions of lexical bundles,” an interesting finding can be observed: the types (Type), tokens (Token), and diversity (TTR) of both the “noun and/or prepositional phrase fragments” structure and the “equivalence” strategy of lexical bundles from professionals are higher than those from trainees, among which significant differences are found in the types (*p*_1_, *p*_2_ < 0.01^9^) and tokens (*p*_1_ < 0.01; *p*_2_ < 0.05) instead of diversity. The significant differences in the types (Type) and tokens (Token) of the structure and strategy between the two groups’ lexical bundles have already been elaborated. But why does the diversity (TTR), unlike the types (Type) and tokens (Token), fail to show any significant inter-group difference? Possible reasons may be that interpreters are limited by time pressure, cognitive load and source text. Firstly, the time to think of diverse bundles for the same meaning is limited in an activity featured by output immediacy. Second, diversity is less likely to be the criterion of quality assessment in an activity with high cognitive load. Interpreting raters tend to focus on information completeness, fluency of delivery, target language quality (idiomatic expression and grammatical correctness) instead of expression diversity (Han and Fan, 2020, p. 113). Third, although professionals, compared with trainees, used more types and frequencies of lexical bundles, the diversity of lexical bundles of professionals still failed to differ significantly from that of trainees, since the types and frequencies of lexical bundles in the ST are fixed. Therefore, the types (Type) and frequencies (Token), except diversity (TTR), of lexical bundles with the “noun and/or prepositional phrase fragments” structure and/or an “equivalence” strategy from professionals are significantly higher than those from trainees.

### Comparing the process of lexical bundles between professionals and trainees

The comparison scope and level in translational relationships between ST and TT have been fully discussed in the section “Types and definitions of structures and strategies of lexical bundles”. Based on rigorous adjustments, reasons for adopting different strategies will be analyzed in the following sections.

#### Reasons for similarities and differences in one-strategy lexical bundles

##### Equivalence

“Equivalence,” referring to that lexical bundles in TT are equivalent to messages in ST, is the most frequently used strategy by both professionals and trainees (P: 61.2%; T: 59.2%, see Table 3). Three structural fragments are all included in the lexical bundles using the “equivalence” strategy from the two groups (P: 74.88% NP/PP, 8.84% DC, 16.28% VP; T: 71.99% NP/PP, 9.93% DC, 18.09% VP^10^). Example (1) contains four four-word lexical bundles (what we need to; we need to do; need to do is; to do is to), all of which belong to the “dependent clause fragments,” corresponding to the ST “我们就是要.”

(1) ST: 下一个阶段 我们就是要想办法让孩子们能够上好学.

*Gloss: In the next stage, we just need to figure out ways to enable children to receive quality education*.

TT: In the next stage, **what we need to do is to** come up with various ways to provide quality education to these children. (P4)

**Figure 2:**
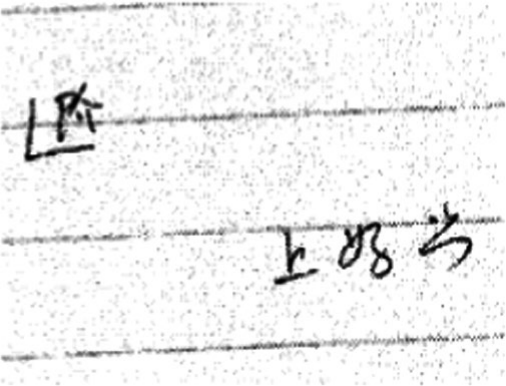
Notes of example (1).

Some lexical bundles may have already been stored as a fixed expression in interpreters’ mind and can be expressed without requiring much cognitive effort. The interpreting product in example (1) shows the lexical bundle is produced accurately and fluently, symbolizing that interpreters barely encountered any difficulty to render it. Additionally, the interpreter reported that s/he did not think much about it when interpreting, and only wrote down “what should be done in this period” in her/his notes. The notes do contain “阶 [period]” and “上好学 [receive quality education]” (see Figure 2), verifying the retrospective protocol. Thus, it can be inferred that the expression “我们就是要” and the lexical bundle “what we need to do is to,” although not noted down, have already been stored in the interpreter’s long-term memory as a pair of equivalents and can be produced without exerting much cognition pressure.

##### Shift

“Shift,” referring to that lexical bundles in TT are substitutive to messages in ST, is a strategy adopted by professionals and trainees with similar frequencies (P:37; T: 35, see Table 3). Three structural fragments are included in lexical bundles using the “shift” strategy (P: 43.24% NP/PP, 37.84% DC, and 18.92% VP; T: 34.29% NP/PP, 42.86% DC, and 22.85% VP). In Example (2), the “noun and/or prepositional phrase fragments” lexical bundle (the development of the) is an alternative to the ST information “办好 [make something successful].”

(2) ST: 刘延东同志在表彰大会上发表了一篇重要讲话，它的题目是“国家发展，希望在教育； 办好教育，希望在教师.”

*Gloss: Comrade Liu Yandong, at the awarding ceremony, delivered an important speech. Its title is “The hope of national development lies in education; the hope of good education lies in teachers”.*

TT1: And he would like to, <uh> his speech was something like this <uh>: the national development of China relies on the education, and **the development of the** Chinese education relies on the teacher faculty’s quality. (P1)

TT2: Now <uh> on the conference <uh> we <uh> we have heard an important talk on the <uh> on the educational business named that <uh> **the development of** the country rely on, **depends on** the quality of education; and **the development of** education **depends on** teachers. (T7)

**Figure 3:**
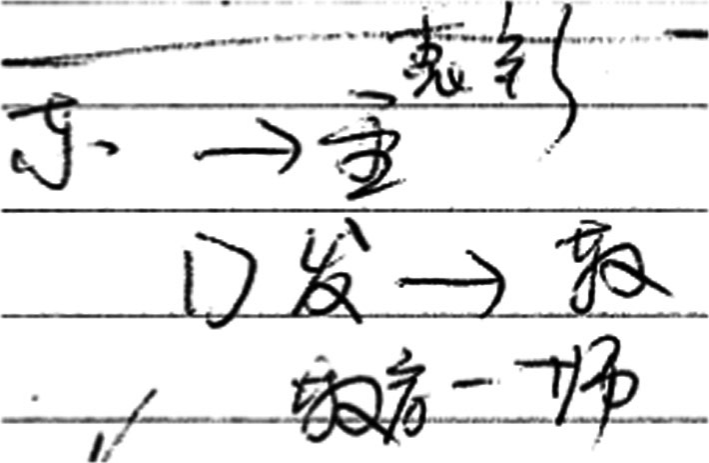
Notes of example (2).

In example (2), the lexical bundle “the development of” may be influenced by the parallel structure of the Chinese title and the “structural priming effect” (Bock, 1986). First, the professional interpreter (TT1) wanted to make the syntactic structure of the TT similar to that of the ST. The rendition (**the** national **development of** China **relies on**…, and **the development of** the Chinese education **relies on**…) matches the parallel structure in the ST (国家发展，希望在…；办好教育，希望在…). But why were “发展 [develop/development]” and “办好[make something successful]” both interpreted as “the development of…”? The answer may be found from the notes (see Figure 3). The combination of “发 [development]” and “—>” in the notes reminds the interpreter of the structure “the development of…relies on…” upon interpreting the first part of the title (国家发展，希望在…). Influenced by the “structural priming effect,” the tendency of reusing a structure appeared before (*ibid*), “the development of… relies on…” was used again in the rendition of the second part of the title (办好教育，希望在…), making the interpretation more parallel in form as well as in meaning. The trainee (TT2) may also be influenced by the two factors and used twice “the development of…depends on…” structure to achieve parallelism. It is hard to render titles well for both professionals and trainees. One possible evidence is that disfluencies can be found before rendering the title in example (2) from both interpreters. Another would be reflected by the retrospection of the professional interpreter (TT1), who mentioned that it is not easy to formulate a proper rendition of the title, so an informal structure “something like this” was inserted to gain more time to think of a suitable equivalent of the title.

##### Addition

“Addition,” referring to that lexical bundles in TT are additional to messages in ST, is more frequently used by professionals than trainees (P: 75; T: 33, see Table 3). Three structural fragments are included in lexical bundles using the “addition” strategy (P: 34.67% NP/PP, 25.33% DC, and 40% VP; T: 48.48% NP/PP, 30.30% DC, and 21.21% VP). Example (3) contains four four-word lexical bundles (to improve the quality; improve the quality of; the quality of the; of the rural teachers), which belong to the “dependent clause fragments,” “verb phrase fragments,” and “noun and/or prepositional phrase fragments” (the last two lexical bundles) respectively. The first three lexical bundles are added information, while the last one is equivalent to the message in ST (the “equivalence” strategy, see the section “Equivalence”).

(3) ST: 我们正在继续努力，一个最重要的措施呢，就是提高农村教 师队伍的经济地位、政治地位、社会地位、职业地位。

*Gloss: We are continuing to work hard, [and] one of the most important measures is to improve rural teachers’ economic status, political status, social status, and professional status.*

TT: However, one of the most important ways, **to improve the quality of the rural teachers’** team, is <~> to improve their economical, their economic, political, social and <uh> professional conditions in the society. (P2)

In example (3), the professional’s three lexical bundles are added information, which is inferable from the context and makes the original information clearer. Such addition, as a kind of explicitation, aims to “clarify the original information” (Tang and Li, 2013, p. 446) as well as “reduce listeners’ processing efforts” (Tang, 2018, p. 50). However, none of the trainees made similar explicitations to “一个最重要的措施 [one of the most important measures].” Possible explanations may be that trainees are more susceptible to “source language shining through” (Teich, 2003, p. 207) and have less efforts and experience to interpret in a more listener-friendly way.

#### Reasons for similarities and differences in two-strategy lexical bundles

##### Equivalence + shift

“Equivalence + shift,” referring to that lexical bundles in TT incorporate both “equivalence” and “shift” strategies when being compared to messages in ST, is more frequently used by professionals than trainees (P: 66, T: 44, see Table 3). Three structural fragments are included in lexical bundles using the “equivalence + shift” strategy in the two groups (P: 42.42% NP/PP, 12.12% DC, and 45.45% VP; T: 54.54% NP/PP, 6.81% DC, and 38.64% VP). The four-word lexical bundle (have a lot of) in example (4) belongs to the “verb phrase fragments,” with “have” corresponding to “有” and “a lot of” replacing “一系列 [a series of].”

(4) ST: 这项措施是根本性的，当然还 有一系列的措施。我们的一个基本思想就是要吸引社会上优秀的人才来当老师，要吸引优秀人才到农村，到基层，去长期从教，终身从教。

*Gloss: This measure is fundamental, [and] of course, there are still a series of measures. One of our basic concepts is to attract talents in society to become teachers, to attract talents to the rural areas [and] to the grassroots, and to work as teachers for a long time or even a lifetime.*

TT: We still **have a lot of** measures concerning this <uh> measure <mea > <uh> program. And we want to <uh> <uh> attract more talents, talents to become teachers, and we want more people to come to the rural area, and to be a teacher <uh> here for a long time. (T4)

The “equivalence” strategy may be used due to an exact memory or information deduction. In example (4), “有[have],” though not being noted down, may have been exactly memorized by the interpreter. Another probability is that the precise rendition of “有[have]” is triggered by “we” as a potential subject in the context and “措[mea(sures)]” as an exact object in the notes, since verbs as “relation” are more likely to be deduced from “argument” served by pronouns or nouns (see the section “Reasons for similarities and differences in structural distributions of lexical bundles,” Ji, 1996; Wang and Zhou, 2014).

**Figure 4:**
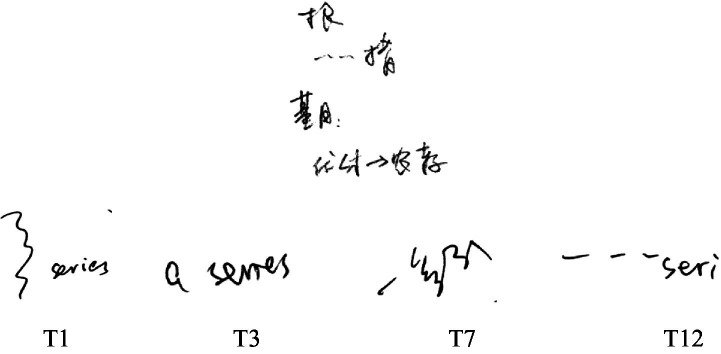
Notes of example (4).

Interpreters tend to adopt the “shift” strategy to deal with unimportant information rather than memorize its exact meaning, so as to allocate their efforts to more important information. In example (4), the “…” in the notes represents “一系列[a series of]” (see Figure 4), but it can also be understood as other similar meanings, like “a lot of” or “quite a few.” Giving up a precise record, the interpreter output an imprecise expression “a lot of,” which deviated a little from the original meaning but did not affect the general meaning. By doing this, the interpreter saved time for formulating more important information that followed. But even with such strategical effort, the following interpretation may still be hard for the interpreter because of a lack of understanding of the notes. The interpreter reflected that s/he was trying to figure out the notes, which could also be evidenced by the recurrent disfluencies (one repair “<mea>” and five hesitations “<uh>”) in the rendition. Nonetheless, the fuzzy processing of non-critical information still partly reduces the cognitive load of interpreters and splits their attention to more important information. Comparing the two groups of interpreters, this study finds that most professionals chose to record “一系列[a series of]” as “﹏” or not to record it at all, while four trainees wrote it down as “︴series,” “a series,” “一系列[a series of],” or “…seri” (see notes: T1, T3, T7, and T12; Figure 4), indicating that professionals are more skillful at simplifying notes and allocating limited cognitive efforts than trainees.

##### Equivalence + addition

“Equivalence + addition,” referring to that lexical bundles in TT incorporate both “equivalence” and “addition” strategies when being compared to messages in ST, is more frequently used by professionals than trainees (P: 89; T: 61, see Table 3). Three structural fragments are included in lexical bundles using the “equivalence + addition” strategy from the two groups (P: 66.29% NP/PP, 14.61% DC, and 19.10% VP; T: 42.62% NP/PP, 19.67% DC, and 37.70% VP). The two four-word lexical bundles (that of the civil, of the civil servants) in example (5) belong to the “noun and/or prepositional phrase fragments.” In the first lexical bundle, “that of” is added information, and “the civil” corresponds to “剬.” The second lexical bundle corresponds exactly to “剬务员的” (the “equivalence” strategy, see the section“Equivalence”).

(5) ST: 在这个要求呢，第一，是要求我们的义务教育的老师，特别 是农村的义务教育的老师的工资收入要不低于当地 **剬务员的** 。

*Gloss: For requirements, firstly, [it] is required [that] our compulsory education teachers, especially rural compulsory education teachers, [their] salary should not be lower than that of local civil servants*.

TT: And through this performance-based salary, we <~> mended that the <~> teachers in <~> the compulsory educational years, especially those in the countryside <country> <uh> in the rural area. <uh> Their salary should not be lower than the, than **that of the civil <uh> servants** of the same area. (P1)

In example (5), “that of” is added for grammatical correctness. The repair (**than the, than that of** the civil <uh> servants) shows that the interpreter initially started with a word-for-word rendition “than the civil servants.” Without finishing the phrase, s/he realized the literal translation was ungrammatical and corrected immediately by restarting the expression and adding “that of” before “the civil servants.”

Furthermore, the “equivalence” strategy is used when it comes to terminology rendition, though with disfluencies. The interpreter in example (5) reflected that his/her mind went blank upon hearing “剬务员[civil servants]” and normally “servants” should be uttered immediately after “civil.” Therefore, disfluencies may appear due to interpreters’ unfamiliarity with terminology or memory lapses led by pressure. The term “剬务员[civil/public servants]” has been interpreted accurately by five professionals and six trainees, while four in the five professionals and two in the six trainees using the “equivalence” strategy rendered it fluently, showing that professionals’ terminology interpretation is better than trainees in fluency instead of accuracy.

##### Shift + addition

“Shift + addition,” referring to that lexical bundles in TT incorporate both “shift” and “addition” strategies when being compared to messages in ST, is less frequently used by professionals and trainees (P: 0.7%; T: 3.6%, see Table 3). Three structural fragments are included in lexical bundles using the “shift + addition” strategy from trainees, while “noun and/or prepositional phrase fragment” is not included in those from professionals (P: 40% DC, 60% VP; T: 47.06% NP/PP, 17.65% DC, 35.29% VP). The four-word lexical bundle (this is a very) in example (6) belongs to the “verb phrase fragments,” among which “this” replaces “绩效考核 [performance appraisal]” and “is a very” belongs to the added information.

(6) ST: 在很多措施当中，最根本的一条，是今年1月1日开始实施 的义务教育教师绩效工资制度…我们进行绩效考核，进一步地 调动广大教师的积极性。

*Gloss: Among many measures, the most fundamental one is the performance-based salary system for compulsory education teachers which was implemented on January 1^st^ this year…We conduct* ***performance appraisals*** *to further mobilize the enthusiasm of teachers.*

TT: We have taken multiple measures. And the most important measure is the, rule of performance-based salary for the teachers of the nine-year <p> compulsory education <p> which was launched in January first… **This is <uh> a very** important measure we have taken, to improve the quality. (P11)

Interpreters use the “shift” strategy to simplify (and at the same time diversify) the already-mentioned information and allocate efforts to more important information that follows. In example (6), “绩效考核[performance appraisal]” can be considered as reoccurred information, for it has a similar meaning to the previous information “绩效工资制度,” which was literally interpreted as “performance-based salary system.” To simplify (and at the same time to diversify) the rendition of the reoccurred message, the interpreter used the pronoun “This” to render “绩效考核[performance appraisal],” so as to allocate efforts to the following information, especially when the following information was difficult for interpreters.

**Figure 5:**
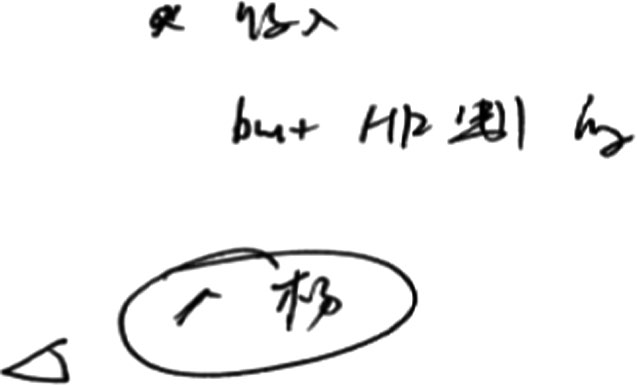
Notes of example (6).

Interpreters may add other ideas as a kind of explicitation to fill the gap caused by “a failure in understanding, remembering or expressing the original message” (Tang, 2018, p.50). In example (6), without noting down “进一步地调动广大教师的积极性[to further mobilize the enthusiasm of teachers]” (see Figure 5), the interpreter might feel difficult to recall the original message, which could be indicated by the hesitation (<uh>) in the rendition. To fill in the gap caused by failure in remembering, the interpreter added “is <uh> a very important measure we have taken” to gain more time for formulating the following ideas. Therefore, the added information as a gap-filling explicitation is a buffer strategy to facilitate information processing and formulation.

#### Reasons for similarities and differences in three-strategy lexical bundles (equivalence + shift + addition)

“Equivalence + shift + addition,” referring to that lexical bundles in TT incorporate “equivalence,” “shift” and “addition” strategies when being compared to messages in ST, is the least used strategy by professionals and trainees (P: 0.1%; T: 0.8%, see Table 3). Lexical bundles with the “equivalence + shift + addition” strategy include “verb phrase fragments” and “noun and/or prepositional phrase fragments” from trainees but only “verb phrase fragments” from professionals (P: 100% VP; T: 75% NP/PP, 25% VP). The four-word lexical bundle (and now we are) in example (7) belongs to the “verb phrase fragments,” among which “and” replaces “但是 [but],” “now we” belongs to added information, and “are” corresponds to “面对着.”

(7) ST: 但是面对着新的形势，其实对我们的教师队伍建设提出了很 高的要求，新的要求。

*Gloss: But facing the new situation, in fact to the construction of our teachers’ team, [it] raised very high requirements [and] new requirements.*

TT: **And now we are** in a new era, so we have new requirements for our teachers and teaching, team. (P3)

**Figure 6:**
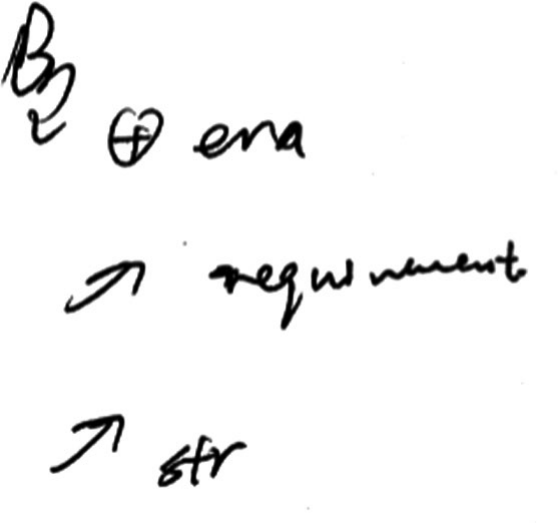
Notes of example (7).

Interpreters need to reorganize the logic or add grammatical components when rendering the original speech, for the colloquial ST is less likely to be rigorous in logic and grammar. On the one hand, interpreters may shift information according to their logic reconstruction even if the rendition may disaccord with the original message or the notes. In the notes of example (7), “B” stands for “But,” consistent with “但是” in the ST (see Figure 6), but it was replaced by “And” in the rendition, indicating that the interpreter did not follow what s/he noted down upon listening to the original message. On the other hand, interpreters may add information inferable from the context to supplement information not appeared in the ST. In example (7), based on the previously-mentioned information “我们已经建立起…[we have established…]” and the following information “新的形势[a new situation/era],” the interpreter can deduce “we” as the subject missed in the ungrammatical sentence and “now” representing the tense (simple present tense).

With the “equivalence” strategy, the verb in the lexical bundle in example (7) was rendered based on information deduction. Verbs as “relation” can be deduced by “argument,” that is, the subject and the object. The subject “we” (and the simple present tense) are deduced from the context. The note “era” stands for the object “a new era” (see Figure 6). Hence, the two “arguments” reminded the interpreter of the “relation” (are) and a reasonable rendition “now we are in a new era” was formulated by the interpreter.

The triangulation (interpretation products, retrospection, and interviews as well as notes) reveals various reasons lying behind strategies in interpreting. The “equivalence” strategy tends to be used because of effort reduction, source language shining through, an exact memory, information deduction, and terminology rendition; the “shift” strategy is inclined to be adopted due to structural priming effect, simplification (and diversification) of expression, cognitive effort allocation, and logic reorganization; the “addition” strategy is likely to be employed owing to explicitation for clarifying information or filling gap and grammatical correctness. Besides, the study finds that longer lexical bundle units (up to nine-word units in this study) are combined by several adjacent four-word lexical bundles, verifying and expanding the finding of “two four-word lexical bundles sometimes occur together to form a five-word or six-word sequence” (Biber et al., 2004, p. 376).

## Conclusion

Given the scarcity of empirical evidence on lexical bundles in interpreting literature, the current study takes an initial step forward, aiming at exploring the similarities and differences in the product and production process of four-word lexical bundles in Chinese-English consecutive interpreting between professional interpreters and trainees. This exploratory research identifies that: (1) for structural distributions, the types (Type) and frequencies (Token) of “noun and/or prepositional phrase fragment” lexical bundles from professionals are significantly higher than those from trainees; (2) for strategical distributions, the types (Type) and frequencies (Token) of lexical bundles using the “equivalence” strategy from professionals are significantly higher than those from trainees; (3) for strategy adoption, both groups may adopt lexical bundles due to common factors including an exact memory, structural priming effect, information deduction, logic reorganization, and grammatical correctness. Professionals are more likely to be motivated by explicitation for clarifying information or filling gaps, cognitive effort allocation and simplification (and diversification) of expression. Trainees are more susceptible to source language shining through and their terminology rendition is better than professionals in accuracy though not in fluency.

Based on the established frameworks tailored for analyzing lexical bundles in interpreting, this study does not simply present their linguistic features (the structural and strategical distributions) like most existing literature but also analyzes reasons behind those features, a new attempt to perceive interpreting product through both description and explanation. Moreover, verified by triangulation based on data collected from different channels (interpreting product, notes, retrospection, and interviews), this study also figures out various factors (e.g., difficulty, quality or language habit) that may drive interpreters’ (professionals’ and/or trainees’) strategy adoption, a new trial to explore interpreting process by transparentizing interpreters’ decision making.

This study may suggest the following directions for future research. Theoretically, the interpreting-tailored lexical bundle frameworks (in terms of structure and strategy) established in this study may be verified, improved or even overturned by future studies. For instance, the two frameworks can be applied to other larger-scale interpreting corpora to test if any exceptions exist. If exceptions do exist, the two frameworks can be partly validated and further improved. Besides, the two frameworks can be applied to interpreting corpora of different language pairs, directions or interpreting modes, which may present totally different lexical bundle taxonomies. Once being overturned in other corpora, the frameworks for lexical bundles in interpreting can be further systematized, with sub-frameworks in different language pairs, interpreting directions or modes.

Methodologically, with disfluencies being tagged, this study may inspire future studies to further explore disfluencies in lexical bundles, including their type, frequency, position, and other factors. In this study, the comparison of the types and frequencies of disfluencies in lexical bundles between professionals and trainees reveals the two groups’ different degree of familiarity with the source information as well as their different ways to cope with unfamiliar expressions upon performing tasks with high cognitive load. In addition, the position of disfluencies in a lexical bundle may not be the exact position where interpreters encounter difficulties. Instead, difficulties may exist before, within or after the lexical bundle with disfluencies. More systematic studies are encouraged to investigate the relationships between the position of disfluencies and the triggering difficulties, an insightful indication of cognitive pressure in interpreting research.

Pedagogically, the differences identified in the linguistic characteristics and strategy adoption of lexical bundles between professionals and trainees as well as their related reasons may raise awareness of the teaching and learning of lexical bundles in interpreting training. It is instrumental for trainers to summarize the gap in the structure and strategy of lexical bundles between professionals and trainees and design targeted exercises to narrow the gap. For trainees, understanding the reasons behind professionals’ strategy adoption may improve their problem solving competence upon conducting interpreting practice. Moreover, with interpreting products, notes as well as retrospection and interviews presented in this study, trainees are able to observe the interpreting product and process of professional interpreters in detail from different perspectives, providing them with a valuable opportunity to learn from professionals.

## Data availability statement

The raw data supporting the conclusions of this article will be made available by the authors, without undue reservation.

## Ethics statement

The studies involving human participants were reviewed and approved by Hong Kong Polytechnic University. The participants provided their written informed consent to participate in this study.

## Author contributions

FT is responsible for designing and conducting the research experiment, analyzing the dataset, and writing the thesis. SJ is responsible for analyzing the data and writing the thesis. All authors contributed to the article and approved the submitted version.

## Funding

This work was supported by the National Social Science Foundation of China (18CYY011).

## Conflict of interest

The authors declare that the research was conducted in the absence of any commercial or financial relationships that could be construed as a potential conflict of interest.

## Publisher’s note

All claims expressed in this article are solely those of the authors and do not necessarily represent those of their affiliated organizations, or those of the publisher, the editors and the reviewers. Any product that may be evaluated in this article, or claim that may be made by its manufacturer, is not guaranteed or endorsed by the publisher.

## Footnotes

^1^Idiosyncratic uses in this study stand for stereotypical idioms, such as *break a leg* (meaning ‘good luck’) and *under the weather* (meaning ‘sick’), or creative expressions coined by interpreters based on the context.

^2^In "ST: 输送更多的高质量的老师; TT: improve the quality of the teachers," "quality" and "teacher" correspond to "质量" and "老师" respectively, while the whole lexical bundle "quality of the teachers" does not correspond to "高质量的老师 [quality teachers]."

^3^Semantic meaning in this article refers to the literal meaning of ST/TT. If the implicit meaning of ST is expressed by interpreters in a lexical bundle in TT, the lexical bundle will be classified into addition (+ other strategy/ies).

^4^The unit of Type, Token or TTR is a four-word lexical bundle.

^5^The larger the TTR is, the higher the diversity of lexical bundles.

^6^"P" represents professionals and "T" trainees in this study.

^7^"Relation" is usually served by verbs or adjectives (Ji, 1996; Wang and Zhou, 2014).

^8^"Argument" is usually served by nouns or pronouns (Ji, 1996; Wang and Zhou, 2014).

^9^p_1_ means p for the "noun and/or prepositional phrase fragments" structure and p_2_ means p for the "equivalence" strategy.

^10^NP/PP stands for "noun and/or prepositional phrase fragments," VP "verb phrase fragments" and DC "dependent clause fragments."
